# Evaluation of speed-accuracy trade-off in a computer task in individuals with cerebral palsy: a cross-sectional study

**DOI:** 10.1186/s12883-017-0920-4

**Published:** 2017-07-27

**Authors:** Deborah Cristina Gonçalves Luiz Fernani, Maria Tereza Artero Prado, Talita Dias da Silva, Thais Massetti, Luiz Carlos de Abreu, Fernando Henrique Magalhães, Helen Dawes, Carlos Bandeira de Mello Monteiro

**Affiliations:** 1 University of West Paulista, Presidente Prudente, SP Brazil; 2 Laboratory Design and Scientific Writing Department of Basic Sciences, ABC Faculty of Medicine, Av. Príncipe de Gales, 821, Vila Principe de Gales, Santo André, SP 09060-650 Brazil; 3 School of Arts, Sciences and Humanities, University of São Paulo, São Paulo, SP Brazil; 4 Post-graduate Program in Rehabilitation Sciences, Faculty of Medicine, University of São Paulo, São Paulo, SP Brazil; 5 Oxford Institute of Nursing and Allied Health Research, Oxford Brookes University, Oxford, UK; 6 Department of Clinical Neurology, University of Oxford, Oxford, UK

**Keywords:** Cerebral palsy, Motor activity, Disabled people, Motor skills

## Abstract

**Background:**

Individuals with Cerebral Palsy (CP) present with sensorimotor dysfunction which make the control and execution of movements difficult. This study aimed to verify the speed-accuracy trade-off in individuals with CP.

**Methods:**

Forty eight individuals with CP and 48 with typical development (TD) were evaluated (32 females and 64 males with a mean age of 15.02 ± 6.37 years: minimum 7 and maximum 30 years). Participants performed the “Fitts’ Reciprocal Aiming Task v.1.0 (Horizontal)” on a computer with different sizes and distance targets, composed by progressive indices of difficulty (IDs): ID2, ID4a and ID4b.

**Results:**

There were no statistical differences between the groups in relation to the slope of the curve (b1) and dispersion of the movement time (r^2^). However, the intercept (b0) values presented significant differences (F(1.95) = 11.3; *p* = .001]), with greater movement time in the CP group compared to the TD group. It means that for individuals with CP, regardless of index difficulty, found the task more difficult than for TD participants. Considering CP and TD groups, speed-accuracy trade-off was found when using different indices of difficulty (ID2 and ID4). However, when the same index of difficulty was used with a larger target and longer distance (ID4a) or with a narrow target and shorter distance (ID4b), only individuals with CP had more difficulty performing the tasks involving smaller targets. Marginally significant inverse correlations were identified between the values of b1 and age (*r* = −0.119, *p* = .052) and between r^2^ and Gross Motor Function Classification System (*r* = −0.280, *p* = .054), which did not occur with the Manual Ability Classification System.

**Conclusion:**

We conclude that the individuals with CP presented greater difficulty when the target was smaller and demanded more accuracy, and less difficulty when the task demanded speed. It is suggested that treatments should target tasks with accuracy demands, that could help in daily life tasks, since it is an element that is generally not considered by professionals during therapy.

**Trial registration:**

ClinicalTrials.gov, NCT03002285, retrospectively registered on 20 Dec 2016.

## Background

Cerebral palsy (CP) is a common cause of motor dysfunction that affects children and adults [1], defined as a non-progressive disorder in the brain that occurs during the fetal period or early childhood [2, 3]. The motor alterations in these individuals yield varying degrees of involvement of the upper limbs [4], impairing functionality and hindering the control and execution of movements [5–8]. Due to these difficulties in motor control, several motor limitations in the upper limbs, such as longer movement durations, reduced trajectory straightness and lower peak velocities, are observed to affect daily activities and participation in several occupational tasks throughout life [9–11].

When considering optimal training programs for individuals with CP, a number of exercise interventions have been considered to be effective including: endurance, strength, speed and power interventions [12–15]. However, there is limited evidence to support the effectiveness of these training approaches for improving mobility and function.

A recent review suggests that speed rather than strength training might be effective in improving functional mobility of CP individuals [13]. However, there is limited understanding as to the relationship between speed and accuracy in individuals with CP. The speed accuracy relationship is particularly important when considering upper limb functional tasks as compared to functional mobility. In typically developed (TD) individuals, movement speed and accuracy, and their relationship, is inversely proportional except when the need for accuracy and speed coexists whereby both can be achieved [16]. For a movement to be performed accurately, muscle coordination and co-contraction need to occur [17, 18]. When a movement is performed quickly, generally a speed modulation strategy is used, whereby muscle co-contraction is reduced with a subsequent reduction in accuracy [16–18].

The mechanisms underlying the speed-accuracy trade-off are more complex than previously thought, with individuals being able to utilize a speed-energy-accuracy trade-off for goal-directed movements; whereby individuals can move faster while preserving movement accuracy by using a strategy where muscles are co-contracted around the joint but at a high energetic cost [16]. As such, a speed modulation strategy is preferred to a co-contraction strategy for faster movements, and a strategy to preserve energy economy typically prevents us from executing faster accurate movements [16].

Considering individuals with CP, changing the distance of a movement may be less taxing, as this can be controlled through a simpler model of agonist and antagonist muscle activity as compared to that required for more accurate movements [19–21]. However, Imms et al. [22] states that spasticity is the most common motor disorder in CP, characterized by a velocity-dependent increase in the tonic stretch reflex which leads to slow and effortful movement. Meskers [23] also pointed out that individuals with movement disorders caused by neurological diseases, such as CP, due to impaired muscle control have atypical force generation and inappropriate tension regulation affecting the normal movement velocity profile, which is essential to obtain fast movements, thus this characteristic could make tasks that demand speed more challenging for people with CP.

Regarding the conflicting evidence about the influence of speed or accuracy demand during a task in individuals with CP, the assessment and investigation of the speed-accuracy trade-off is essential in order to develop optimal exercise interventions that consider the speed at which movements should be performed when trying to facilitate accuracy and the components of a task (distance, size of the target). Task manipulation may for instance increase difficulty and require individuals with CP to move slower and with greater neuromuscular cost. Perhaps more importantly a better understanding will inform the design of systems and devices for individuals with CP, such as modification of computer switch systems to integrate considerations of distance and target accuracy in order to improve and ease engagement in occupational tasks.

In order to better understand the relationships between distance and accuracy, participants were engaged in a movement time task with different indices of difficulty (IDs). The same ID was performed in two ways (considering the relation between widths and distances), where tasks were performed with thicker sidebars and longer distances between them and thinner sidebars with shorter distances, in order to evaluate if the size of the target (accuracy demand) has more influence in the performance than the distance between them (speed demand).

Given this, this study aimed to verify the speed-accuracy trade-off in individuals with CP. We hypothesize that there should be an inverse relationship between task difficulty and performance (measured by means of movement time) for both TD and CP individuals, whereby the CP individuals should be generally slower than the TD controls and this effect would be more pronounced during movements with higher levels of difficulty. We further hypothesized that the greater the severity of CP, the greater should be the impairment in performance, but with no differences between tasks with same index of difficulty, i.e. tasks that demand more accuracy (size of target) or speed (distance between targets).

## Methods

### Study design

An observational study that analyzed the motor control system by evaluating the performance in a computer task in individuals with CP and in a control group of typically developed individuals.

### Participants

In total, 96 individuals were evaluated, being 48 individuals with CP and 48 with typical development (TD), matched by age and sex. The sample was composed of 32 females and 64 males with a mean age of 15.02 ± 6.37 years (minimum 7 and maximum 30 years). The participants of the CP group comprised 25 individuals with hemiparesis, 19 with diparesis and 4 with quadriparesis. A previous statistical analysis was made to find out differences between type of CP, considering the indices of difficulty as dependent variables, with no difference found (*p* > 0.05). Thus, we include all participants in the study, wherein all individuals in the CP group showed to be able to perform the task.

In the Gross Motor Function Classification System (GMFCS) [24], 29 individuals were classified with level I, 6 with level II, 6 with level III and 7 with level IV. According to the Manual Ability Classification System (MACS) [25], 34 individuals obtained level I, 9 level II and 5 level III.

The inclusion criteria were a medical diagnosis of CP, of levels I to IV according to the GMFCS, a classification system that ranks individuals with CP from I to V, in which children in Level I present some difficulty with speed, balance, and coordination, but with ability to perform all the activities of their age-matched peers, while children in level V present difficulty in achieving any voluntary control of movement and controlling their head and trunk posture in most positions [26]. For the inclusion criteria, they should also be between levels I to III according to MACS, that similarly to GMFCS, but considers upper limb function specifically and consists of five levels, where level I represent the highest functional level and level V the lowest functional level [25]. These classification were made by two professionals specialized in CP. The last inclusion criterion was previous use of mouse in their computational activities.

The exclusion criteria were the presence of surgery or a chemical neuromuscular blockade in the upper limbs within 6 months prior to participation in the study and disorders in cognitive function that would prohibit comprehension of the experimental instructions. The comprehension ability was determined through two trials to perform the task, with lack of comprehension being determined on individuals not attempting to follow instructions and perform the task.

### Material

The software used in this study was "Fitts' Reciprocal Aiming Task v.1.0 (Horizontal)" developed by Okazaki [27], in the public domain and available on the Internet, which was performed on a Toshiba notebook®, model Satellite A60-S1561, Fortrek® OM-302.

This instrument is used to verify motor control through analysis of the speed and accuracy of movement, which is determined through the log-linear relation between target size and task distance between them using a mathematical equation, and analyzed by Fitts’ law, which describes the relation between movement accuracy and speed [28], resulting in the difficulty index. And, the more difficult the task, this will require greater movement time for execution [29]. Thus, the ID2 was composed by target sidebars thicker (3 cm), with little distance between them (6 cm) – (*Log2 [(2 × 6)/3] = Log2 4 = 2*), the ID4a had thicker sidebars (3 cm) with a greater distance between them (24 cm) – (*Log2 [(2 × 24)/3] = Log2 16 = 4*), and the ID4b had thinner target sidebars than used in ID4a (1.5 cm), however, the with less distance between them (12 cm) – (*Log2 [(2 × 12)/1.5] = Log2 16 = 4*) [30, 31].

The task was composed of targets of different sizes, being that the smaller targets require more time to execute due to the necessity of increased accuracy [32, 33] and, if the distance between targets reduces, the speed of movement becomes greater and the accuracy decreases.

**Fig. 1:**
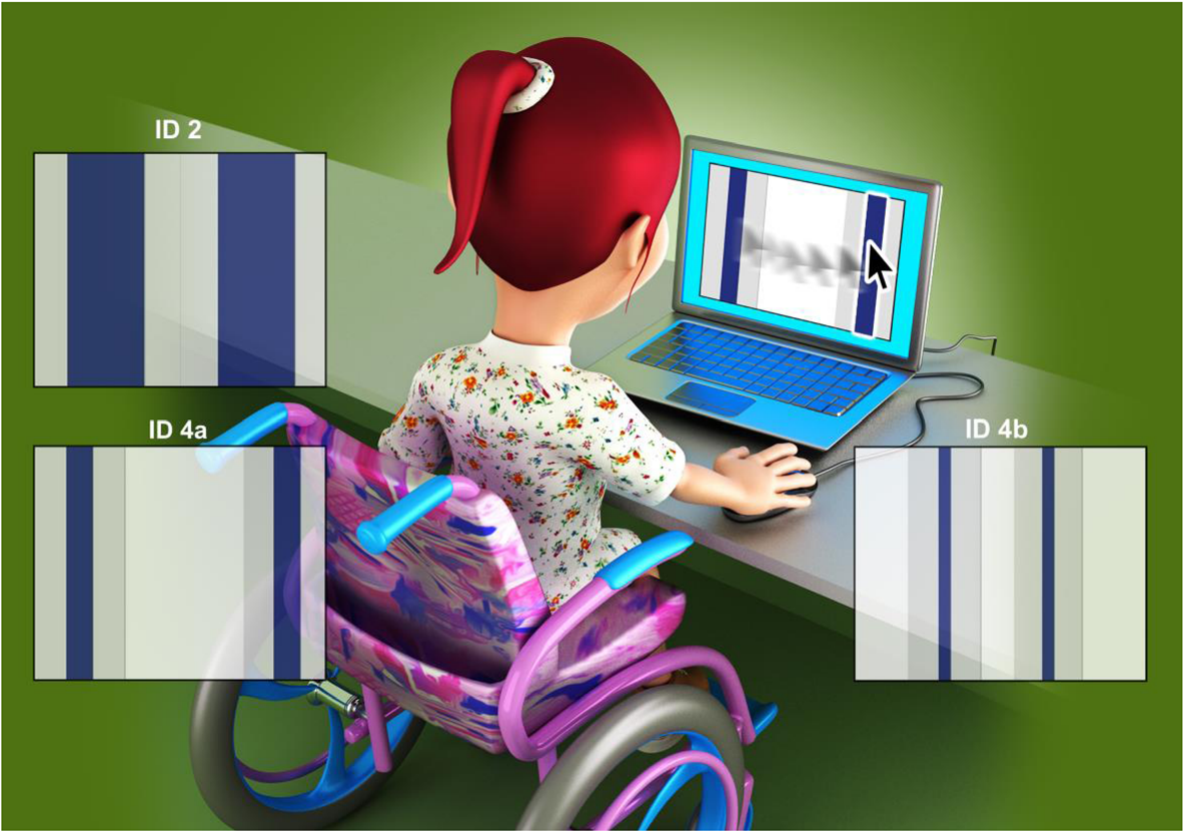
Individual in a wheelchair* performing the task interfaces at different indices of difficulty *some individuals in this study were seated on a regular chair. Source: figure created by the authors

Therefore, to evaluate the trade-off between speed and accuracy, two different indices of difficulty were used (ID2 and ID4). The difficulty level was increased by changing the width and distance between the bars (Fig. 1). In addition, to evaluate if the size of the target (accuracy demand) requires more movement time when compared to the distance between them (speed demand), ID4 was used in two different ways (ID4a and ID4b), for which the distance between the bars and the width were different, but the ID was maintained. As the ID was maintained, should not exist difference in movement time between ID4a and ID4b.

### Procedure

The experiment was composed of three trials at each of the indices of difficulty: ID2, ID4a and ID4b. The participants performed the tasks individually in a room, with only the evaluator present, seated on a chair (or their own wheelchair), which was adjusted in height according to the needs of the individual. A footrest was available, when necessary. The computer was placed on a table, and each participant was given instructions and presented with the task, in which the individual, after hearing an alarm from the computer, was required to click alternately and intermittently with an external mouse cursor on two parallel bars with three sizes of target and three distances which were used to determine different indices of difficulty, and arranged vertically. The instructions given were ‘on hearing the alarm click with the greatest speed and accuracy possible, for a period of 10 seconds, until you hear a second alarm which indicates the end of your attempt’.

Directly following the attempt, the total movement time was registered by software, dividing the seconds obtained in each attempt by the number of “clicks” on targets. If more than two clicks were wrong, the individual repeated the task.

We did not assess or give instructions about the pattern of movement, so they could perform the task in the easier way for each one, i.e. movement of the upper limb in general (hand, wrist, elbow, and shoulder).

### Statistical analysis

Regarding the comparisons for movement time on each index of difficulty, an ANOVA with factor 2 (groups: Cerebral Palsy - CP, Typical Development - TD) by 3 (index of difficulty - ID: ID2, ID4a, ID4b) was conducted, with repeated measures for the factor index of difficulty. Post hoc test was carried out using Tukey-HSD (*p* < .05).

In order to establish whether the groups responded to the relation between accuracy and speed, a linear regression analysis was performed on the movement time data, using the curve estimation and obtaining the values of: b0 (intercept), b1 (slope of the curve) and r^2^ (movement time dispersion) of each participant; every attempt at the indices of difficulty being considered. MANOVA One way was used to compare the means of the groups in the three variables of interest (b0, b1 and r^2^).

A multiple linear regression analysis considering increase in movement time (∆ between 4a and 2; and between 4b and 4a) was performed to determine which factors (age, gender, Gross Motor Function Classification System - GMFCS, Manual Ability Classification System - MACS) influenced the increase in movement time.

To verify associations between GMFCS, MACS, age, b0, b1 and r^2^, the Pearson correlation coefficient was used. The software package used was SPSS, 20.0.

## Results

In this study it was verified that the CP group used longer movement time to complete the task than TD group. An inverse relationship was observed between speed and accuracy of movement in individuals with TD, but not in those with CP.

The ANOVA with factors groups (CP and TD) and index of difficult (ID2, ID4a, ID4b), with repeated measures for the factor ID was performed to explore the effects of index of difficult on the movement time, determined in items below.

### ID2 – ID4a

There was a statistically significant difference between the indices of difficult [F(1.94) = 69.3; *p* < .001; ŋ^2^ = 0.42]. This result demonstrates that the movement time increased significantly from ID2 (mean = 1019 ± 60 ms) to ID4a (mean = 1449 ± 60 ms). However, no interaction was found between Groups and indices of difficult.

In addition, an effect for groups [F1.94) = 33.9; *p* < 0.001; n^2^ = 0.27] was found, in which the individuals with CP (mean = 1575 ± 80 ms) were 682 ms slower than those in the TD group (mean = 893 ± 80 ms).

### ID4a – ID4b

There was a statistically significant difference between the indices of difficult [F(1.94) = 4.62; *p* = .034; ŋ^2^ = 0.05] and interaction between index of difficult and Groups [F(1.94) = 6.85; *p* = .010; ŋ^2^ = 0.07]. This result demonstrates that the movement time increased significantly from ID4a (mean = 1449 ± 60 ms) to ID4b (mean = 1614 ± 6 ms). However, this increase was significant in the CP group from ID4a (mean = 1791 ± 92 ms) to ID4b (mean = 2157 ± 154 ms), while in the TD group the increase was not significant (mean = 1107 ± 92 ms for mean = 1071 ± 154 ms, respectively).

The difference between groups remained [F(1.94) = 29.7; *p* < .001; ŋ^2^ = 0.24]; the individuals of the CP group (mean = 1974 ± 115 ms) were 885 ms slower than the TD group (mean = 1089 ± 115 ms).

These findings suggest that this group with CP did not total obey the relation between size and distance of targets (see Fitts’ law). This observation becomes evident when comparing the results of ID4a and ID4b, which should have presented the same difficulty. However, we found that movement time increased significantly from ID4a to ID4b in individuals with CP, while in the TD group this difference did not occur, respectively). Interestingly, this result is controversial to our hypothesis that there would be no differences between ID4a and ID4b.

**Fig. 2:**
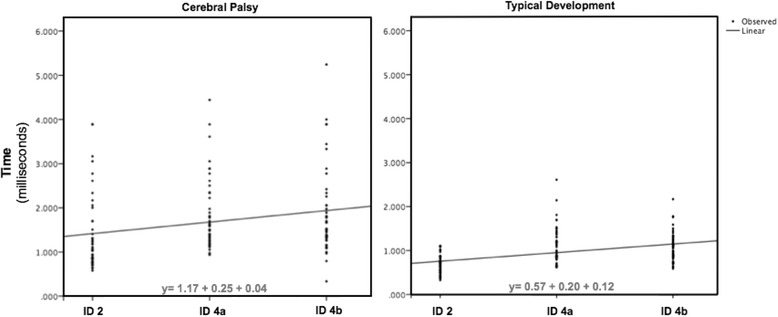
Linear regression analysis using values b0, b1 and r^2^

MANOVA was used to compare the slope of the regression line (Fig. 2) and there was no statistical difference between the groups in relation to the slope of the curve (b1) or between the dispersion of movement time (r^2^) data between the groups. However, the b0 (intercept), values presented statistically significant differences [F(1.95) = 11.3; *p* = .001], with a greater movement time in the CP group [mean = 1150 ± 1100 milliseconds (ms)] compared to the TD group (mean = 560 ± 70 ms).

Marginally significant inverse correlations were identified between the values of the slope of the curve and age and between the dispersion of movement time and GMFCS (Table 1) using the Pearson correlation, which did not occur with the MACS.

**Table 1:** Pearson’s correlation between the values of b0, b1 and r^2^, age, GMFCS and MACS

**Variables**	**Age**		**GMFCS**		**MACS**	
	*r*	*p*	*r*	*p*	*r*	*p*
b0	−0.035	0.737	0.022	0.880	0.151	0.306
b1	−0.119	0.052*	0.216	0.141	0.129	0.384
r^2^	0.083	0.421	−0.280	0.054*	−0.224	0.125

GMFCS, Gross Motor Function Classification System; MACS, Manual Ability Classification System; *marginally statistically significant difference

The linear regression analysis revealed a significant finding, F(6, 41) = 2.44, *p* = 0.041, r2 = 0.263, resulting in the following equation: increase in movement time (ID4a – ID2) = 0.045 × GMFCS. In other words, only the GMFCS influenced the increase in movement time, but age, gender and MACS did not contribute. To understand which factors influenced the increase in movement time from ID4a to ID4b, another regression analysis was performed between difference between ID4a and ID4b with age, gender, GMFCS and MACS, but there were no significant findings for the regression model.

## Discussion

The majority of functional manual skills upper limb require fast and accurate performance which we observed was affected in individuals with Cerebral Palsy (CP), who demonstrated significantly greater movement time regardless of the task difficulty when compared with typically developed individuals. For individuals with CP, a typical payoff between speed and accuracy was found, except importantly that they presented with more difficulty performing tasks involving smaller targets than when moving longer distances. Our findings are important as for the first time we can specifically evidence both therapy and device us in this group.

Considering this specific motor control difficulty, and the associated higher neuromuscular cost for individuals with CP, particularly those with more severe disability, we propose therapists should consider the difficulty performing dexterous tasks that involve fine control, and implement adaptations to increase target size in particular when aiming to reduce neuromuscular demand in occupational situations. Our findings inform therapeutic exercises and the design of a number of occupational tasks for example in the optimal design of communication computer switch systems.

In this study it was verified that the CP group used longer movement times to complete the tasks than did the typical developed (TD) group. An inverse relationship was observed between speed and accuracy of movement in individuals with TD but not in those with CP. These findings, as presented before, are contrary to our hypothesis and suggest that this group with CP did not obey the relation between size and distance of targets (see Fitts’ law). This observation becomes evident when comparing the results of ID4a and ID4b, which should have presented the same difficulty. However, we found that movement time increased significantly from ID4a (mean = 1791 ± 92 ms) to ID4b (mean = 2157 ± 154 ms), in individuals with CP, while in the TD group this difference did not occur (mean = 1107 ± 92 ms for mean = 1071 ± 154 ms, respectively).

These results partially corroborate the findings of Smits-Elgesman [34], who affirmed that individuals with CP require more time to perform the task. The authors also reported that both CP and control groups responded similarly to increasing indices of difficulty, in agreement with the relation between speed and accuracy. This was only partially observed in our present study: in fact, based on our present findings, when observing results from ID2 compared with ID4 there is an influence between speed and accuracy. In ID2 both groups had a smaller movement time compared with ID4. However, considering ID4a versus ID4b individuals with CP had more problems with accuracy (narrow targets, ID4a) than speed (more distant targets, ID4b), this is probably due to weakness, altered reflexes and difficulties in body coordination, that lead to impaired postural control [35, 36].

Davies [4] also found evidence of individuals with CP not following the relation between speed and accuracy. These authors stated that the International Organization for Standardization indicates that tasks requiring accuracy, such as a point-and-click task, present a low ID if less than 4 and a high ID if greater than 6. However, the study of Davies [4] showed that the ID for individuals with bilateral CP should be limited to a maximum of ID2, which could also be associated with the different performance of CP individuals between the ID4a and ID4b found in this study.

Michmizos [31] reported that the analysis of movement time is an indicator of neurological integrity. In this context, delays in movement time have been seen in studies that evaluated individuals with CP [37, 38], in agreement with that shown in the present study, in which was noted that individuals with CP began to execute the task with a movement time greater (mean = 1150 ± 1100 ms) than the TD group (mean = 560 ± 70 ms).

Considering the slope of the curve (b1), the CP group did not differ from TD group in the present study, which demonstrates that the individuals with CP evolved during the attempts, similarly to those with TD, although the group with CP maintained greater movement time for all levels of difficulty compared to the TD. This can be confirmed by the analysis of the performance differences in the indices of difficulty, in which greater time of movement was observed from ID2 to ID4a for both groups.

Both groups did not differ also regarding dispersion of movement time (r^2^) in the evaluated groups; however, both groups presented r^2^ values less than 1, which means that there was variation in movement time values. Although, with no significance, the CP group presented greater variation during the execution of attempts than the TD, verified by a lower value of dispersion of movement time (see Fig. 2). These findings (i.e., the lower movement speed and larger dispersion of the data of the CP group), have also been reported in several studies [4, 34, 39–41], which further suggests a decline in the motor control system associated with impairments that affect both nervous and muscular structures. Furthermore, there is evidence from previous studies that movement time is much larger in individuals with CP than in those with TD [42–45].

The correlation analyses performed to investigate the association between the task measurements and MACS, GMFCS and age (made by Pearson coefficients) showed that there was no correlation between MACS and age, intercept, slope of the curve and movement time dispersion. This finding can be explained by the sample classification (levels I, II, III), as they were able to manipulate the mouse and adequately perform the task. This was also emphasized by Davies [46], who related that individuals with levels I, II and III are able to use a mouse to access a computer, thus the MACS was not sensitive to this relation. In addition, Eliasson [25], described that approximately 65% of individuals with CP are classified as levels I to III of MACS, a fact that confirms the levels used in this study.

On the other hand, there was a significant negative correlation between slope of the curve and age, movement time dispersion and GMFCS. These findings demonstrate that the higher the age of the individual, the less the slope of the curve, i.e. the less time this individual would need to perform the task (older individuals were less sensitive to increases in indices of difficult), and the higher the level of the GMFCS, the lower the value of movement time dispersion (i.e. higher the level of the GMFCS, greater the dispersion of the movement time data). As well as the regression analysis showed that the GMFCS influenced the increase in movement time from ID2 to ID4a, i.e., the higher the GMFCS, the greater the increase in movement time.

With respect to impairment in motor function, studies such as those by Haak [47] and Hanna [48], reported that there is a decline in motor function into adulthood in individuals with severe CP, which can be explained by decreased activity, increased body size and changes in spinal alignment, therefore, it is believed that the better performance displayed by older individuals in the present study was due to higher levels of motor function, i.e. we did not evaluated severe levels, GMFCS V and MACS IV and V.

Thus, to improve the motor performance in individuals with CP which targets the performance of activities of daily living, it is necessary to construct and adapt rehabilitation therapies, including tasks that involve speed and accuracy demands [31]. Moreover, as with CP individuals had larger movement times for increased accuracy demands as compared to increased speed it is suggested that treatments should target accuracy demands, an element that is generally not considered by professionals during conventional therapy, which often emphasizes stretching and strengthening exercises.

### Limitations and future studies

A limitation of this study is, that we were unable to explore the relationships of motor (spasticity and range of motion), visual or sensory functioning to speed and accuracy data; the impact of these variables should now be explored in an appropriately powered study. The variation of the topographic type, MACS and GMFCS levels can be considered vies in this study, however the sample represents the distribution of impairment in this population. In addition, we did not assess kinematic features of the movement to strength our results, as well as we did not evaluate more indices of difficulties, so we believe that further studies in CP should insert these evaluations as additional analysis considered alongside presenting symptoms.

## Conclusions

Individuals with CP presented greater difficulty when the target was smaller and demanded more accuracy, and less difficulty when the task demanded speed. It is suggested that treatments should target accuracy demands, that could help in daily life tasks and is an element that is generally not considered by professionals during therapy.

Thus, therapists should be made aware of this difficulty performing tasks that involve fine control, and consider implementing adaptations to increase target size in particular when aiming to reduce neuromuscular demand in occupational situations, with appreciation the training of functional activities that address the motor control with focus on the evolution of accuracy of movement.

Our results advise the need to implement precision training in therapies and therefore the adaptation of computer systems to integrate considerations of distance and target accuracy in order to improve and ease engagement in occupational tasks.

## Abbreviations

b0: Intercept
b1: Slope of the curve
CP: Cerebral palsy
GMFCS: Gross motor function classification system
ID: Index of difficulty
ID2: Index of difficult 2
ID4a: Index of difficult 4a
ID4b: Index of difficult 4b
MACS: Manual ability classification system
r^2^: Dispersion of the movement time
TD: Typical development
